# The Impact of Rate Adaptation Algorithms on Wi-Fi-Based Factory Automation Systems

**DOI:** 10.3390/s20185195

**Published:** 2020-09-11

**Authors:** Tommaso Fedullo, Federico Tramarin, Stefano Vitturi

**Affiliations:** 1 Department of Management and Engineering, University of Padova, S. S. Nicola 3, 36100 Vicenza, Italy; tommaso.fedullo@phd.unipd.it; 2 Department of Engineering “Enzo Ferrari”, University of Modena and Reggio Emilia, 41125 Modena, Italy; 3 National Research Council of Italy, CNR–IEIIT, Via Gradenigo 6/B, 35131 Padova, Italy; stefano.vitturi@ieiit.cnr.it

**Keywords:** wireless industrial networks, rate adaptation, IEEE 802.11, factory automation, real-time networks

## Abstract

Factory automation systems based on the IEEE 802.11 Wi-Fi standard may benefit from its Multi-Rate Support (MRS) feature, which allows for dynamically selecting the most suitable transmission rate for the targeted application context. The MRS is implemented by means of rate adaptation algorithms (RAAs), which has already demonstrated to be effective to improve both timeliness and reliability, which are typically strict requirements of industrial real-time communication systems. Indeed, some of such algorithms have been specifically conceived for reliable real-time communications. However, the computational complexity of such algorithms has not been effectively investigated yet. In this paper, we address such an issue, particularly focusing on the execution times of some specific rate adaptation algorithms, as well as on their impact on the automation tasks. In this respect, after a formal description of the algorithms, we present the outcomes of an extensive experimental session, which includes practical measurements and realistic simulations. The obtained results are encouraging, since the measured execution times indicate that rate adaptation algorithms can be profitably adopted by industrial automation systems, allowing for improving their reliability and timeliness without impacting on the overall performance.

## 1. Introduction

Modern factory automation systems are based on distributed architectures in which communication networks are exploited to connect industrial controllers and field devices, like sensors and actuators, located throughout a plant, possibly at considerable distances [1]. During operation, the components of these systems execute tasks (e.g. data acquisition and analysis, control, data fusion, etc.), which have to be appropriately scheduled to achieve the expected plant behavior [2,3]. In this scenario, task execution times represent key variables, mainly determined by two components. The first one is concerned with the local (internal to a device) elaboration, which typically comprises the CPU time that is necessary to execute the protocol stack code, as well as the latencies due to data buffering and queuing. The second one is related to the communication times necessary for transmitting data to the components distributed over the plant. Thus, for example, the instance of a control task in a networked system comprises, not only the time necessary to execute the control algorithm on a local controller, but also both the time necessary to acquire the feedback data, obtained from a message sent by remote sensors connected to the network, and the time to send control commands to remote actuators, again via the network [4]. Communication times play a critical role in task scheduling, since their values can be substantially higher than those due to local elaboration and, more importantly, they may be strongly variable. A typical cause of such variability is represented by a failure in delivering a packet, which necessarily requires a retransmission: in this case, the actual task communication time results at least doubled with respect to the time necessary to a single transmission attempt. This problem is even more evident when wireless networks are used, since they are inherently error prone [5,6].

The usage of wireless technology is of strategic importance to enable the Industrial Internet of Things (IIoT), which claims for the connection of industrial equipment (things), making them reachable from anywhere, at any time, using any service [7]. IIoT is part of the Industry 4.0 plan, which is fostering a significant transformation within the field of industrial automation and control. Some of the leading issues of this paradigm are represented by the need of interconnection, integration, and interoperability among possibly heterogeneous industrial systems [8].

The IEEE 802.11 Wireless LAN [9], universally known as Wi-Fi, already demonstrated its ability to cope with the tight requirements of the industrial field of application [10]. Moreover, it is easy to use, flexible, and able to provide the required interoperability [11]. Additionally, Wi-Fi has an attractive feature, namely the Multi–Rate Support (MRS), which revealed to be definitely advantageous in environments subjected to frequent fluctuations of the Signal–to–Noise Ratio (SNR), such as the industrial ones [12]. Indeed, the use of suitable Rate Adaptation (RA) Algorithms (RAAs) allows to dynamically change the packet transmission rate to better cope with the channel status. Roughly speaking, the most common RAAs select low transmission rates for low values of the SNR, since lower rates adopt more robust modulation schemes.

Several RAAs have been designed over the years and some of them were specifically conceived for industrial applications [12,13]. These latter ones have demonstrated their effectiveness, in that they are able to rapidly adapt to the SNR behavior, and revealed to be able to ensure low communication times, along with less variability and limited packet loss ratios. However, the computational complexity and execution times of such algorithms have not been effectively addressed yet. This is an aspect of considerable importance which may have a serious impact on the overall tasks execution times, especially when devices with limited resources, such as industrial sensors/actuators are used.

This paper moves from the above considerations and focuses on the computational aspects of rate adaptation algorithms, as well as of protocol stacks, adopted for industrial real–time applications, as will be described in the following.

## 2. Related Work and Contribution

The actual impact of task execution times on the performance of distributed automation systems has only rarely been addressed by the scientific literature. More commonly, theoretical analyses and/or experimental measurements on prototype systems have been carried out. In this respect, an essential review of related works, mostly concerned with Wi-Fi systems, is provided in the following.

In [14], the authors provide an interesting survey that addresses the timeliness of the IEEE 802.11 MAC mechanisms. Another survey is given by [10], where the authors focus more specifically on the deployment of Wi-Fi for time critical and reliable industrial control applications. In [15], the authors investigate the adoption of frequency–domain based access protocols to improve the performance of legacy Wi-Fi. In [16], the features of IEEE 802.11ax are analyzed in detail to achieve efficient scheduling (i.e., an effective assignment of users to subcarriers in order to improve network throughput). The design and practical implementations of real–time networks based, to different extents, on IEEE 802.11, are provided in [17,18], respectively. In [17], the authors propose a new MAC protocol based on a Time Division Multiple Access (TDMA) technique that relies on the IEEE 802.11 Distributed Coordination Function (DCF), capable of providing deterministic performance. A prototype implementation of the protocol actually demonstrated its effectiveness. In [18], a modification of the IEEE 802.11 physical layer is introduced in order to achieve very low packet delivery times, which were comparable with those of the wired counterparts. In [19], the authors describe WIA–FA, a communication system for factory automation based on Wi-Fi, standardized by the International Electrotechnical Commission (IEC) [20], along with some interesting case studies and examples of application to real manufacturing plants. Real–time industrial multimedia applications, particularly video streaming, are addressed in [21], where the authors show the capability of Wi-Fi to handle such a kind of traffic. A valuable example of calculation of protocol stack execution times is given in [22]. Here, the authors not only describe a possible measurement procedure, but also provide the results of some experimental sessions aimed at evaluating the execution times of both the TCP/IP and UDP/IP protocol stacks for a given embedded device.

The aforementioned studies represent interesting and valuable contributions that, however, do not address the real behavior of devices, typically determined by non-negligible computational requirements and times that may reveal of paramount importance in the real-time industrial communication context [23]. Focusing on Wi-Fi, this aspect might be exacerbated by the adoption of RAAs that, undeniably, require an undefined amount of time for the execution of their code. Hence, there is the risk that the benefits achieved with the adoption of MRS on the communication side, in particular the reduced packet delivery times, are vanished by the possible increase of the local elaboration times, caused by the execution of the rate adaptation algorithms. These considerations fostered the preparation of this paper whose main contribution can be summarized, as follows:

- Implementation of rate adaptation algorithms for industrial real-time applications and experimental assessment of their execution times;
- Evaluation of the impact of rate adaptation algorithms on the protocol stacks execution times and on the communication times;
- Performance assessment of Wi-Fi–based industrial communication networks with rate adaptation algorithms enabled.

In detail, the paper is structured, as follows. Section 3 provides some essential background about real–time industrial networks. Section 4 presents the rate adaptation algorithms conceived for real-time communication, to give the foundations to evaluate the impact of their execution times. Section 5 describes the implementation of the algorithms and introduces the setup adopted for the experimental sessions. Section 6 presents the tests that were executed and discusses the obtained results. Finally, Section 7 concludes the paper and outlines some future research activities.

## 3. Background: Real–time Industrial Networks

Real-time industrial networks have been extensively addressed by the scientific literature [1,24]. They are communication systems that are specifically designed to effectively interconnect industrial devices, such as controllers, sensors, and actuators. This type of communication implies the timely transmission of limited amounts of data (some tens of bytes, typically) carrying, for example, set–points values or process data variables. Industrial networks have often to comply with tight timing requirements due to the peculiarity of the applications for which they are deployed. Indeed, they have to grant for the precise scheduling of periodic messages, as well as to ensure that aperiodic messages are delivered within a-priori specified deadlines. Notably, packet transmission periods and deadlines can be as low as some hundreds of microseconds. The introduction of wireless networks in this challenging context needed careful analyses, due to the well known problems that may affect wireless communication, such as fading, path loss, and collisions, which inevitably lead to delays in packet delivery or, even worse, to packet losses [25,26]. This topic has been addressed by several research activities in the past years, that led to the latest state of play in which wireless networks are widespread in the industrial scenario, particularly for process automation applications [27,28]. Moreover, their deployment is expected to grow significantly in the next future [29].

Suitable protocols have been adopted in order for both types of industrial networks (i.e., wired and wireless) in order to fulfill the aforementioned requirements. The most widespread are based on techniques, like TDMA (Time Division Multiple Access), Master–Slave, and Producer–Consumer [30,31]. In general, these protocols introduce an ordered access scheme to the transmission medium that allows for properly handling the message flows generated by the applications. For example, in a Master–Slave configuration, if a temperature sensor (the Slave) has to transmit its value with a given periodicity to a controller (the Master), then it will be periodically granted by the Master with the right to access the network to transmit the temperature value. In this way, the desired timing behavior can be achieved. Additionally, with such protocols packet collisions are definitely reduced, since stations are not allowed to initiate a transmission autonomously and, hence, to transmit contemporaneously.

## 4. Rate Adaptation Algorithms for Wi–Fi–Based Real Time Industrial Networks

The MRS feature of IEEE 802.11 can be deployed to dynamically change the transmission rate of a packet. Indeed, the adopted rate can be selected from a set of available rates before each transmission.

**Definition** **1** (Set of available rates)**.** *The set of the available rates, R, for the transmission of a packet is defined, as follows.*

$$R=\left\{r_{1},r_{2},...,r_{n}\right\}\;where\;r_{i}>r_{i-1},\;i\in \{1,2,...,n\}.$$

Basically, the operation of a RAAs consists in the selection of an ordered set of rates, called Retransmission Chain, to be used for the transmission of a packet.

**Definition** **2** (Retransmission chain)**.** *Consider the transmission of a packet for which a maximum of N attempts can be carried out. The Retransmission Chain, RC, is defined as*

$$RC=\left\{r^{\left(1\right)},r^{\left(2\right)}...,r^{\left(N\right)}\right\},\;r^{\left(i\right)}\in R.$$

*meaning that the first transmission attempt is scheduled at rate $r^{\left(1\right)}$, the second at rate $r^{\left(2\right)}$, the N-th at rate $r^{\left(N\right)}$*

### 4.1. ARF: Automatic Rate Fallback

Automatic Rate Fallback (ARF) [32] is one of the most popular rate adaptation strategies for Wi-Fi, designed for general purpose applications.

The behavior of ARF can be briefly described, as follows. Consider a set *R* of available data rates. Starting from the generic rate $r_{i}$, a station implementing ARF moves to the lower rate $r_{i-1}$ after *K* consecutive failed transmission attempts. Conversely, the rate is increased to $r_{i+1}$ after *N* successful attempts. Also, if a failure occurs at the first transmission attempt after the rate has been increased, then the rate is immediately lowered down to $r_{i-1}$ (this feature is known as "Probing transmission"). Typical values of the parameters *K* and *N* for the ARF algorithm are 2 and 10, respectively [33].

Unfortunately, ARF demonstrated to be rather ineffective for real–time industrial communication. However, its behavior allowed to derive two new algorithms able to reduce both the number of retransmissions and the delivery time of a packet, that revealed suitable for the industrial scenario. These are, namely, Static retransmission rate ARF (SARF) and Fast rate reduction ARF (FARF) [12].

### 4.2. SARF: Static Retransmission Rate ARF

SARF [12] is a variant of ARF in which each retransmission attempt is carried out at rate $r_{1}$, i.e., the lowest rate in the set *R*. In practice, if the first transmission attempt fails, then for all the subsequent retransmissions we will have $r^{\left(i\right)}=r_{1}$ $\forall r^{\left(i\right)}$ ∈RC with i∈{2,3,...,N}. However, a successful retransmission carried out at the lowest rate is not considered as an event that resets the consecutive failures counter in order to grant SARF with the same sensitivity to channel variations of ARF. More precisely, only the original (first) transmission attempt is considered, and if *K* of these attempts fail then the rate will be lowered anyway, even if they were interleaved by one or more successful retransmissions. SARF ensures a very high success probability in packet transmission, since the lowest transmission rate uses the most robust modulation. This allows to limit the number of retransmissions, with the consequent reduction of the randomness. The pseudo-code of SARF is described by Algorithm 1.

**Algorithm 1** SARF Algorithm
 1:/* *Initialization* 2:ccf←0;           ▹ Number of consecutive transmission failures 3:ccs←0;        ▹ Number of consecutive transmission successes 4:$R\leftarrow \{r_{1},r_{2},...,r_{n}\};$                ▹ Available rates 5:i←1;           ▹ Select the lowest transmission rate in set *R* 6:$tr\leftarrow r_{1};$           ▹ Set transmission rate to the lowest value 7:/* *End Initialization* 8:
**while true do**
 9:    **if** Request of a new transmission **then**10:        **if** Previous attempt was failed **then**11:           Perform transmission attempt at the lowest rate12:        **else**13:           Perform transmission attempt at the current rate14:           **if** Success **then**15:               ccf←0;            ▹ Reset # of consecutive failures16:               ccs++;       ▹ Increase counter of consecutive successes17:               **if** ccs≥N**then**          ▹ If success threshold reached18:                   **if** i<n**then**            ▹ If highest rate not reached19:                       i++;20:                       $t_{r}\leftarrow r_{i};$                     ▹ Increase rate21:                   **end if**22:               **end if**23:           **else**24:               /* *(If failure)*25:               ccs←0;           ▹ Reset # of consecutive successes26:               ccf++;              ▹ Increase consecutive failures27:               **if** ccf≥K**then**          ▹ If failure threshold reached28:                   **if** i>1**then**             ▹ If lowest rate not reached29:                    i−−;30:                       $t_{r}\leftarrow r_{i};$                   ▹ Decrease rate31:                   **end if**32:               **end if**33:           **end if**34:        **end if**35:    **end if**36:
**end while**



### 4.3. FARF: Fast Rate Reduction ARF

FARF is an algorithm that represents a customization of ARF in which K=1 and $r^{\left(i+1\right)}\leftarrow r_{1}$. In practice, just a single failed attempt is sufficient to set the new transmission rate to the lowest value in the set *R*, (i.e., $r_{1}$). Conversely, the increasing of the rate follows the same rules of ARF, and happens after *N* consecutive successful attempts. Analogously to SARF, $r^{\left(i\right)}=r_{1}$ $\forall r^{\left(i\right)}\in RC$ with i∈{2,3,...,N}. From a certain point of view, FARF is a more conservative algorithm than SARF, since it intrinsically assumes that a failure is an event that requires to maintain low rates for a certain number of subsequent packet transmissions. The pseudo-code for FARF is described by Algorithm 2.

**Algorithm 2** FARF Algorithm
 1:/* *Same code as SARF Algorithm (lines 3 to 7)* 2:
**while true do**
 3:    **if** Request of a new transmission **then** 4:        Perform transmission attempt 5:        **if** Success **then** 6:             /* *Same code as SARF Algorithm (lines 16 to 22)* 7:        **else** 8:             /* *(If failure)* 9:             ccs←0;         ▹ Reset # of consecutive successes10:           $t_{r}\leftarrow r_{1};$    ▹ Set transmission rate to the lowest value11:        **end if**12:    **end if**13:
**end while**



### 4.4. RSIN: Rate Selection for Industrial Networks

RSIN is a rate adaptation algorithm specifically conceived for the industrial environment, proposed in [13], and based on two fundamental assumptions. Specifically, (i) a station wishing to send a frame (of a given length) has to be aware of the SNR perceived by the receiver and (ii) the station has to know the relationship between SNR and packet error rate (PER) for all the rates of the transmission set *R*. For each packet to be transmitted, let *D* be its deadline. Subsequently, the outputs of RSIN are the number of retransmissions ($N_{opt}$), the retransmission chain ($RC_{opt}$) and the residual error rate probability for the transmission of that packet in a time less than *D*. More formally, RSIN can be described by a constrained optimization problem.

**Definition** **3** (RSIN constrained optimization problem)**.** *For each packet transmission let be:*

*D*  the transmission deadline;
*s*  the signal–to–noise ratio;
*l*  the payload of the packet to transmit; and,
$N_{max}$ the maximum number of retransmission attempts.

*Let us define:*

*N*  the number of allowed retransmission attempts;
$P_{r}$ the residual packet transmission error probability, given N retransmissions at rates $r^{\left(1\right)}....r^{\left(N\right)}$; and,
$t_{trans}$ the transmission time.

*Subsequently, the constrained optimization problem is to find an optimal number of retransmissions attempts $N_{opt}$, and a retransmission chain $RC_{opt}=\left\{r_{opt}^{\left(1\right)},r_{opt}^{\left(2\right)}...,r_{opt}^{\left(N_{opt}\right)}\right\}$, which minimize the residual packet transmission error probability, while ensuring that the deadline is not missed. That is*

(1) $$\left\{\begin{array}{c} \min_{N\leq N_{max},r^{\left(i\right)}}P_{r}\left(l,s,N,RC\right)\\ \max_{N\leq N_{max},r^{\left(i\right)}}t_{trans}\left(l,s,N,RC\right)\leq D,\;i=1\cdots N \end{array}\right.$$

RSIN is described by Algorithms 3 and 4. One of the core sections of Algorithm 3 is represented by lines 18–20. At this stage, a vector of retransmission chains is built, and each element stores the chain ensuring the lowest transmission error probability for that specific number of retransmission attempts, ranging from 1 to $N_{max}$. This is achieved with the invocation of Algorithm 4. Subsequently, lines 22–33 select the optimal retransmission chain among the $N_{max}$ chains of the ${\bar{RC}}_{low}$ vector, as the one associated with the lowest transmission error probability. It is worth noting, in Algorithm 4, how the selection of the retransmission chain is handled in case more retransmission chains ensure the same transmission error probability. As can be seen (lines 9–13), in this case Algorithm 4 selects the retransmission chain that has the lower transmission time. A further assumption has been adopted to limit the execution time of RSIN. Specifically, the construction of the array of retransmission chains by Algorithm 4, has been carried out under the condition

(2) $$r^{\left(1\right)}\geq r^{\left(2\right)}\geq ...\geq r^{\left(N\right)}$$

that limits the retransmission chains to be considered.

**Algorithm 3** RSIN Algorithm
 1:
*N*

▹ number of retransmission attempts
 2:/* ***Inputs*** */

 3:
$N_{max}$

▹ max number of retransmission attempts
 4:
$R=\{r_{1},r_{2},...,r_{n}\}$

▹ set of available rates
 5:
*D*

▹ Packet deadline
 6:
*s*

▹ SNR value
 7:/* ***Definitions*** */

 8:
$t_{trans}$

▹ packet transmission time
 9:
$P_{r,opt}$

▹ residual error probability for the optimal rate sequence
10:/* **Definitions – Vectors of size $N_{max}$** */

11:
${\bar{P}}_{r}$

▹${\bar{P}}_{r}\left[i\right]$: residual error probability when $N=i,\;i=1\cdots N_{max}$
12:
${\bar{t}}_{trans}$

▹${\bar{t}}_{trans}\left[i\right]$: transmission time when $N=i,\;i=1\cdots N_{max}$
13:
${\bar{RC}}_{low}$

▹ Vector of rate chains.
14:/* The i-th element, ${\bar{RC}}_{low}\left[i\right]$, contains the retransmission chain of length N=i that ensures the lowest transmission error probability*/

15:/* ***Outputs*** */

16:
$N_{opt}$

▹ optimal number of retransmissions
17:
$RC_{opt}=\left\{r_{opt}^{\left(1\right)},r_{opt}^{\left(2\right)},...,r_{opt}^{\left(N_{opt}\right)}\right\}$

▹ optimal retransmission chain
18:**for**i←1 to $N_{max}$ **do**

19:    Running Algorithm 4.

20:
**end for**


21:/* Calculation of $N_{opt}$ and $RC_{opt}$ */

22:
$N_{opt}\leftarrow 1$


23:
$RC_{opt}\leftarrow {\bar{RC}}_{low}\left[1\right]$


24:
$P_{r,opt}\leftarrow {\bar{P}}_{r}\left[1\right]$


25:**for**i←2 to $N_{max}$ **do**

26:    **if** ${\bar{P}}_{r}\left[i\right]<P_{r,opt}$ **then**

27:        $N_{opt}\leftarrow i$

28:        $RC_{opt}\leftarrow {\bar{RC}}_{low}\left[i\right]$

29:        $P_{r,opt}\leftarrow {\bar{P}}_{r}\left[i\right]$

30:    **else**

31:        Do nothing

32:    **end if**

33:
**end for**



**Algorithm 4:** Selection of the retransmission chain ensuring the lowest transmission error probability for each value of *N*
1:
**repeat**
2:    Generate a retransmission chain of size *i*, $RC_{i}$3:    calculate $t_{trans}\left(l,s\in S,N,RC_{i}\right)$4:    **if** $t_{trans}\left(l,s\in S,N,RC_{i}\right)\leq D$ **then**5:        **if** $P_{r}\left(l,s\in S,N,RC_{i}\right)<{\bar{P}}_{r}\left[i\right]$ **then**6:           ${\bar{t}}_{trans}\left[i\right]\leftarrow t_{trans}\left(l,s\in S,N,R_{i}\right)$7:           ${\bar{P}}_{r}\left[i\right]\leftarrow P_{r}\left(l,s\in S,N,R_{i}\right)$8:           ${\bar{RC}}_{low}\left[i\right]\leftarrow RC_{i}$9:        **else if** $P_{r}\left(l,s\in S,N,R_{i}\right)={\bar{P}}_{r}\left[i\right]$ **then**10:           **if** $t_{trans}\left(l,s\in S,N,RC_{i}\right)<{\bar{t}}_{trans}\left[i\right]$ **then**11:               ${\bar{t}}_{trans}\left[i\right]\leftarrow t_{trans}\left(l,s\in S,N,RC_{i}\right)$12:               ${\bar{P}}_{r}\left[i\right]\leftarrow P_{r}\left(l,s\in S,N,R_{i}\right)$13:               ${\bar{RC}}_{low}\left[i\right]\leftarrow RC_{i}$14:           **else**15:               Do Nothing16:           **end if**17:        **else**18:           Do Nothing19:        **end if**20:    **else**21:        **Continue**22:    **end if**23:**until** there are sequences of rates $r_{j}$, of length i,∈R, meeting condition 2



Finally, in [34] a variant of RSIN, namely RSIN with Estimation (RSIN–E), has been introduced. RSIN–E has been devised to address the cases in which the SNR is not made available by the receivers. Thus, a learning algorithm has been designed to provide an estimation of the SNR, as better detailed in [34]. The learning algorithm is executed cyclically, with update period $T_{U}$. Clearly, the introduction of a further algorithm has the effect of increasing the execution time of RSIN–E, as will be investigated in the following of the paper. In particular, the lower the update period, the higher the impact on the execution time.

## 5. Algorithms Implementation and Experimental Set–Up

**Figure 1:**
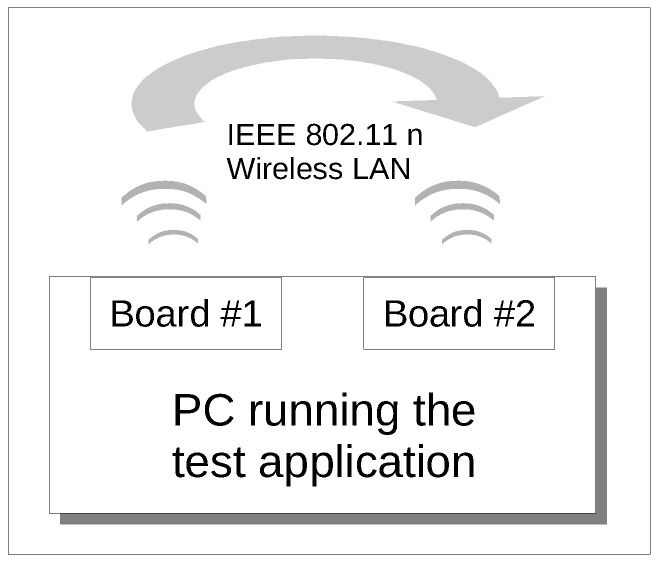
A Sketch of the Experimental Set–up.

The RAAs described in the previous section, with the exclusion of ARF, have been practically implemented on a Personal Computer (Dell Optiplex 960) equipped with an Ubuntu Long Term Support Linux distribution, based on Linux kernel version 4.1.0-040100-lowlatency. The PC is equipped with two commercial off-the-shelf Wireless Network Interface Cards (WNICs), namely TP-LINK model TL-WN851ND, in order to implement an IEEE 802.11n network, as described in Figure 1. The used WNICs are based on the AR9287 chip, which adopts the open–source ath9k driver. This choice, along with adequate modifications to the mac80211 module, following the technique outlined in [35], allowed to implement and test the designed rate adaptation algorithms.

Clearly, the use of a PC with two WNICs does not reflect a real industrial network set-up at all. However, the first set of experiments we executed do not need to be carried out on an actual industrial network, as it will be better detailed in the following. Indeed, the performed analysis is aimed at evaluating the execution times of the RAAs and their impact on the MAC layer execution times. Such times are related to the algorithms implementation and do not depend on the network configuration. Moreover, having the two WNICs installed on the same PC revealed definitely advantageous, because it allowed for exploiting a unique clock source for the experiments, ensuring high accuracy of the results, since there was no need to synchronize the two boards.

For the specific case of the RSIN algorithm, we developed two different implementations. In the first one, the constrained optimization problem (Equation (1)) is run before the transmission of each packet. In the second implementation, referred to as “RSIN Light” (RSIN–L) in the following, a set including all of the optimal retransmission chains, for each possible SNR value, is determined at an early initialization phase, and is stored within a look-up table. Thus, the selection of the retransmission chain, which happens just before a single packet transmission, simply involves access to that table. This strategy clearly reduces the execution time of the algorithm. However, because the constrained optimization problem is not solved dynamically, RSIN–L implicitly assumes that (i) the relationship between SNR and PER does not vary over time, (ii) the deadline is the same for all the transmitted packets, (iii) the packets have fixed length. The actual assessment of such conditions depends on the communication channel status as well as on the specific application.

The choice of using a Personal Computer for the implementation of the algorithms, as well as of the subsequent measurement campaign, has been suggested by the flexibility ensured by such device. Indeed, it has been possible to adequately modify the MAC layer protocol stack, as well as to enable/disable and tune the rate adaptation algorithms during the execution of the tests. However, in real applications different devices may be used, since factory automation systems make use of Programmable Logic Controllers (PLCs), sensors/actuators and, possibly, Soft PLCs. All of these devices, typically, do not grant full access to the MAC layer functions and, hence, could have not been used for our purposes. Nevertheless, and more importantly, rate adaptation algorithms do exist and are (or may be) used by such kind of devices. In this respect, we believe the results of the practical experiments we carried out, which will be discussed in the next Section, represent a meaningful indication of the impact of rate adaptation algorithms on the performance of industrial devices.

**Figure 2:**
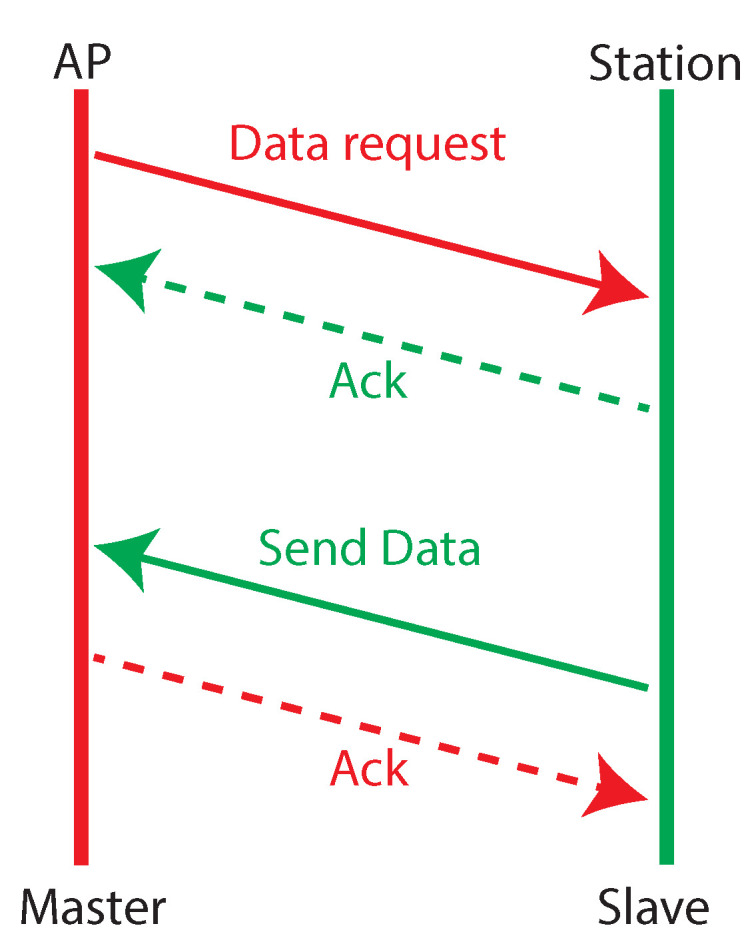
Data transaction between the two Wireless Network Interface Cards (WNICs).

The RAAs have been implemented within the context of the Linux mac80211 framework. In agreement with this approach, we developed the set of required functions for each algorithm. A test application has been subsequently designed, in which the data exchange described in Figure 2 was triggered periodically, thus resembling a typical industrial polling sequence. A Master–Slave relationship has been set-up where one board (the Master), configured as Access Point, sends a request packet to the other one, configured as Station (the Slave), which responds with a data packet. Each transaction ends when the Master receives the packet with the requested data. The complete list of the settings used for the experiments is summarized in Table 1. They have been selected to comply with the most common features/requirements of Wi-Fi based real-time industrial networks and their relevant rate adaptation algorithms, as described in [25,36,37,38].

**Figure 3:**
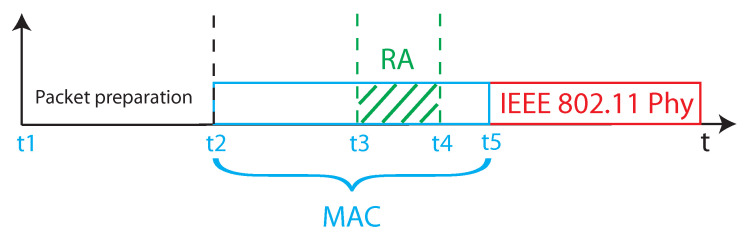
Timings related to the transmission of a packet.

We introduced adequate timestamp points within the relevant parts of the protocol stack code in order to measure the RAAs execution times. Figure 3 provides a representation of the timings related to the transmission of a packet. As can be seen, the packet is initialized at time $t_{1}$ and delivered to the MAC layer at time $t_{2}$. The execution of the RAA takes place in the interval $t_{3}-t_{4}$. The packet is eventually delivered to the physical layer at time $t_{5}$. Notably, the reference clock shared by the two WNICs allowed to precisely timestamp the events concerned with code execution.

## 6. Performance Assessment

### 6.1. Execution Times of Rate Adaptation Algorithms

**Figure 4:**
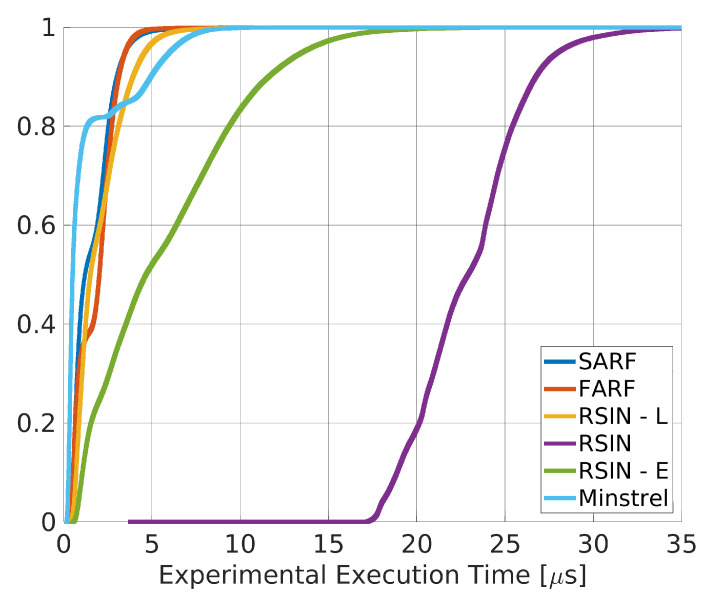
ECDFs for the comparison of the RAAs execution times.

The execution time of each rate adaptation algorithm has been measured placing timestamps at the instants $t_{3}$ and $t_{4}$ in Figure 2. The obtained statistics are given in Table 2, whereas Figure 4 reports the Experimental Cumulative Distribution Function (ECDF).

As can be seen, the statistics also comprise Minstrel, a widespread general purpose algorithm [35]. Minstrel has not been designed for real–time industrial applications. However, it has been included here as an effective basis for comparison. From the presented results, it appears evident that SARF, FARF and RSIN–L have execution times of the same order of magnitude of Minstrel. Conversely, RSIN has the highest execution time, as an effect of the constrained optimization problem algorithm executed before each packet transmission. RSIN–E has an intermediate value, due to the choice of using a look up table in the experiments for such algorithm, analogously to RSIN–L. A higher value is expected in case the constrained optimization problem is executed before each packet transmission. Also, the mean execution time of RSIN–E can be reduced by increasing the update time of the learning algorithm, $T_{U}$, which in this experiment was set to a rather low value (10 ms, as reported in Table 1).

In industrial applications, in order to assess real–time performance, often worst case communication times have to be taken into consideration. For this reason, we calculated the maximum execution times of the algorithms, that are reported in Table 3. As can be seen SARF, FARF and RSIN–L perform better than Minstrel, whereas both RSIN and RSIN–E show higher times, in agreement with the previous analysis.

### 6.2. Impact of the Rate Adaptation Algorithms on the MAC layer Execution Time

**Figure 5:**
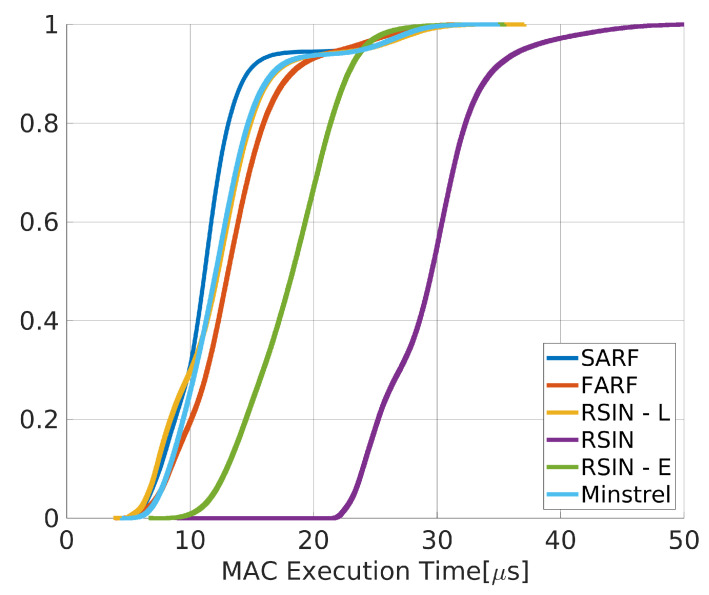
ECDFs representing the IEEE 802.11 MAC layer execution times.

In a further session of experiments, we determined the execution times of the MAC protocol with the rate adaptation algorithms running, and evaluated the relevant impact of the algorithms themselves on such times. The *impact* is defined as the percentage of MAC layer execution time used to run the RA algorithm, and expressed as the ration between the algorithms execution time and the overall execution time of the MAC protocol stack. The MAC layer execution time has been measured, with refer to Figure 3 as the difference between the two timestamps $t_{2}$ and $t_{5}$. The statistics are reported in Table 4, whereas Figure 5 shows the ECDFs curves. The Table also reports the impact of RAAs.

As can be seen, the experimental results reveal that the impact of the rate adaptation algorithms on the IEEE 802.11 MAC layer execution times may be considerable, depending on the selected algorithm.

### 6.3. Impact of the Rate Adaptation Algorithms on Communication

In order to achieve deeper insights about the various components of a data exchange cycle, we evaluated the time taken by the communication sequence of Figure 2, that in the following we refer to as round trip time (RTT), and the consequent impact of the rate adaptation algorithms on it. Similarly to the previous section, the impact is evaluated as the percentage of RTT used to run the RA algorithm. In this experiment, we decided to use the same network configuration shown in Figure 1 that, as already observed, does not reflect that of a typical industrial network. Nonetheless, the obtained results are meaningful. Indeed, the proximity of the two WNICs ensures an efficient communication, characterized by the selection of high transmission rates by the RAAs and very limited packet retransmissions, as we assessed in a post processing analysis. Consequently, the communication times resulted definitely lower than those achievable with a (more realistic) distributed industrial network. Thus, the measured RTT represents a lower bound for such a performance index. Conversely, the measured impact is an upper bound, since the execution times of the rate adaptation algorithms do not depend on the network configuration.

**Figure 6:**
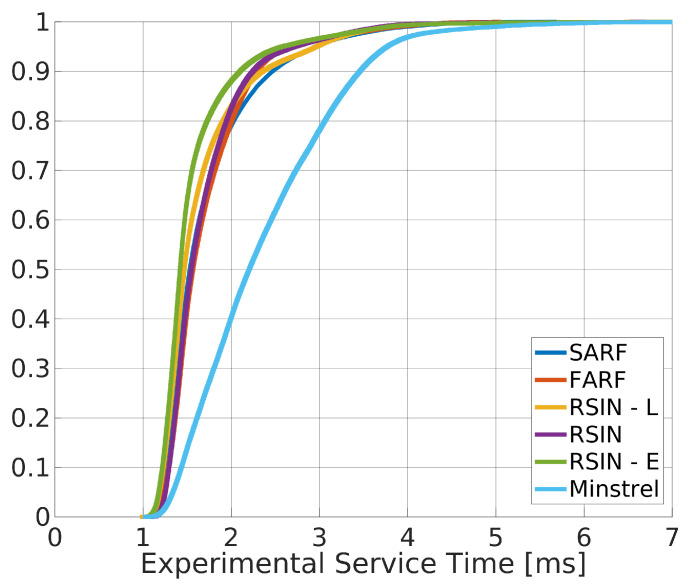
ECDFs for the comparison of the RTT when rate adaptation is enabled.

The RTT has been calculated inserting two timestamps in the code, the first one placed at time $t_{1}$ (in Figure 3), and the second one, with refer to Figure 2, at the instant of data reception from the Slave. The resulting ECDFs are presented in Figure 6.

At a first glance, from the figure, it appears evident that the adoption of rate adaptation algorithms specifically designed for real–time communication algorithms results definitely advantageous, since they allow to achieve low values of round trip times, characterized by very limited variability, as also confirmed by the statistics reported in Table 5. This is due to the very high probability of success at the first transmission attempt they achieve (for example, we measured 98.2% for SARF and 99.6% for RSIN). In this way, the random backoff times between two subsequent transmission attempts were mostly avoided, with the consequent benefits on timeliness [13].

More importantly, Table 5 shows that the impact of the execution time of all the rate adaptation algorithms on RTT is very limited, in particular, also for algorithms such as RSIN and RSIN–E that showed longer execution times than the other ones. This result on the one hand confirms that the adoption of the rate adaptation algorithms is advantageous whereas, on the other hand, it shows that communication times are predominant over elaboration times.

### 6.4. Minimum Cycle Time for a Wi–Fi based Real–time Industrial Network

In a final session of tests, we carried out a set of simulations to investigate the impact of the execution times on the minimum cycle time (MCT), a typical performance index for industrial networks [1,39]. The MCT has been introduced for networks based on a cycle (that are widespread in the industrial scenario) in which a master device regularly polls a set of slaves. In such a kind of configurations, MCT is defined as the minimum time employed by the master to subsequently poll all the slaves.

We referred to industrial network configurations typically deployed at the lower levels of factory automation systems, such as production islands, that comprise a controller and a limited number of sensors/actuators [40]. Also, the parameters selected for this test are those listed in Table 1. For the sake of simplicity, aperiodic traffic has not been considered. In order to execute realistic simulations, we took into consideration the results of the previous measurement sessions. In particular, the measured MAC layer execution times were introduced in the simulation model of all the involved nodes. Moreover, from the communication point of view, we adopted an approach similar to that presented in [41,42]. Transmission and Reception correlation matrices are calculated to simulate a multi-path MIMO channel. Specifically, we used the “F” channel model, proposed by the IEEE 802.11 Task Group n, that is targeted for industrial environments. The simulation setup comprised one master and a variable number of slaves. The polling of a slave, as described in Figure 2, starts with a data request packet issued by the master and ends with the reception of the response data from the slave. In the simulations, we considered two rate adaptation algorithms among those addressed so far, namely RSIN and RSIN–L. Furthermore, as a basis for reference, we plotted the behavior of the MCT for the (purely theoretical) case in which the execution time of the rate adaptation algorithm was not considered. This is referred to as “No–ET”. The settings of the network were those presented in Table 1.

**Figure 7:**
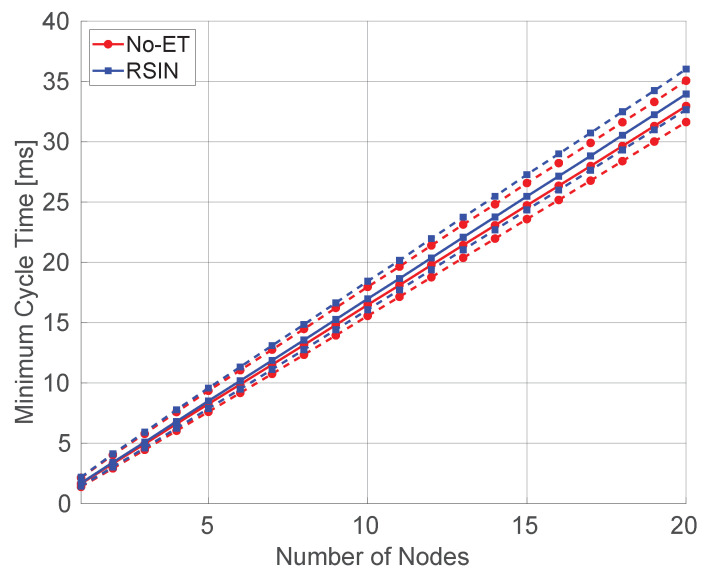
Minimum Cycle Time for a Prototype Industrial Network.

The behavior of the MCT versus the number of nodes is provided in Figure 7 that reports the mean values (continuous lines) and the variation range relevant to the 5th and 99th percentiles, respectively (dotted lines). The statistics are reported in Table 6. To ensure good readability the figure does not comprise RSIN–L that, however, has a behavior very close to that of No–ET. As can be seen, although the adoption of RSIN leads to a greater MCT, with respect to No–ET, its impact is rather limited. As an example, for the case of 20 nodes, it results 2.94%. These outcomes are confirmed by the statistics of MCT, which also show that the variability introduced by the rate adaptation algorithm on the MCT is negligible. As a final remark, it may be observed that, practically, the MCT increases linearly with the number of nodes. This is not surprising, because the main cause of non–linearity is represented by packet retransmissions during the polling of slaves, that introduce random backoff times, with the consequent negative impact on the MCT. A packet needs to be retransmitted either when it incurs in a collision or when it is lost for other communication impairments. However, with the adopted Master–Slave protocol, collisions are negligible (slaves can not transmit contemporaneously) and so are the consequent packet retransmissions. Moreover, packet retransmissions due to other causes are limited by RSIN, that cleverly selects transmission rates able to ensure the best packet transmission success probability.

## 7. Conclusions and Future Directions of Research

In this paper, we addressed the impact of rate adaptation algorithms on the task execution times of distributed automation systems, based on Wi-Fi. The analysis has been carried out gradually, via practical experiments as well as by simulations. We first evaluated the execution times of the algorithms, then we determined their impact on the MAC protocol stack and, eventually, on the task execution times. The obtained results clearly indicate that the adoption of rate adaptation algorithms in real-time industrial communication systems should be encouraged, since they bring considerable benefits in terms of timeliness and reliability with a limited impact on the task execution times and, hence, on industrial control scheduling policies.

Future activities may be envisaged in two directions. The first one involves the development of new rate adaptation algorithms for the industrial scenario, and/or the enhancement of the existing ones. To this regard, the behavior of RSIN could be made more effective, in order to further limit its execution time. For example, some Retransmission Chains that are not able to meet a deadline could be a priori excluded, reducing in such a way the number of iterations performed in Algorithm 3.

The second direction refers to the execution of more comprehensive experimental assessments. Particularly, measurements should be carried out on real industrial devices, in order to achieve a complete characterization of their behavior.

## Figures and Tables

**Table 1:** Settings of the experimental testbed.

Parameter	Value
Period of Transactions	10 ms
Number of transactions	100,000
Packet Length	50 bytes
K_SARF_	2
N_SARF_ = N_FARF_	10
Update Period RSIN-E, T_U_	10 ms
RSIN and RSIN-E Deadline	2 ms
Center Frequency	2660 MHz
Channel model	IEEE 802.11n
Channel bandwidth	40 MHz
STBC	Enabled
LDPC	Disabled
Guard Interval	Normal
N of spatial streams	2
Available Data rates [Mbps]	13.5, 27, 40.5, 54, 81, 108, 121.5, 135

**Table 2:** Execution time statistics of the rate adaptation algorithms.

Algorithm	Mean	Std. deviation
SARF	1.56895 μs	0.993964 μs
FARF	1.80424 μs	0.999401 μs
RSIN-L	1.95316 μs	1.24508 μs
RSIN	22.8111 μs	2.9518 μs
RSIN-E	5.5637 μs	3.98806 μs
Minstrel	1.29763 μs	1.79429 μs

**Table 3:** Maximum values of the execution time of the selected RAAs.

Algorithm	Max Value
SARF	4.81206 μs
FARF	4.33086 μs
RSIN-L	6.13538 μs
RSIN	31.7115 μs
RSIN-E	17.2933 μs
Minstrel	7.71133 μs

**Table 4:** Impact of the RA techniques on the MAC layer execution time.

	MAC Execution Time		
Algorithm	Mean	Std. deviation	Impact [%]
SARF	11.7137 μs	6.26242 μs	13.39
FARF	13.4602 μs	6.81398 μs	13.40
RSIN-L	12.5359 μs	6.9672 μs	15.58
RSIN	29.4723 μs	7.00321 μs	77.40
RSIN-E	18.096 μs	6.90356 μs	30.75
Minstrel	12.7162 μs	6.91258 μs	10.20

**Table 5:** Statistics of the Round Trip Time and Impact of the RA techniques.

	Round Trip Time		
Algorithm	Mean	Std. Deviation	Impact [%]
SARF	1.69607 ms	0.978333 ms	0.1836
FARF	1.6906 ms	0.965661 ms	0.2134
RSIN-L	1.61245 ms	0.927467 ms	0.2423
RSIN	1.66003 ms	0.891203 ms	2.7478
RSIN-E	1.53468 ms	0.789313 ms	0.7250
Minstrel	2.26982 ms	1.47906 ms	0.1143

**Table 6:** Statistics of the MCT and Impact of the RA algorithms.

Nr.	RSIN		RSIN–L		No–ET	
Nodes	Mean	Dev.	Mean	Dev.	Mean	Dev.
	[ms]	[ms]	[ms]	[ms]	[ms]	[ms]
5	8.5064	0.4198	8.3022	0.4241	8.2566	0.4274
10	16.9741	0.5772	16.5751	0.5879	16.4748	0.5883
20	33.9502	0.8226	33.1381	0.8314	32.9521	0.8386
